# Monthly transthoracic echocardiography in young adults for 6 months following SARS‐CoV‐2 infection

**DOI:** 10.14814/phy2.15560

**Published:** 2023-01-03

**Authors:** Sophie S. Osada, Rachel E. Szeghy, Nina L. Stute, Valesha M. Province, Marc A. Augenreich, Andrew Putnam, Jonathon L. Stickford, Abigail S. L. Stickford, Gregory J. Grosicki, Stephen M. Ratchford

**Affiliations:** ^1^ Department of Health & Exercise Science Appalachian State University Boone North Carolina USA; ^2^ Department of Cardiovascular Medicine Northwest Health – Porter Valparaiso Indiana USA; ^3^ Biodynamics and Human Performance Center Georgia Southern University (Armstrong) Savannah Georgia USA

**Keywords:** cardiac structure and function, cardiac troponin‐I, COVID‐19, transthoracic echocardiography

## Abstract

Severe acute respiratory syndrome coronavirus 2 (SARS‐CoV‐2) can elicit acute and long‐term effects on the myocardium among survivors, yet effects among otherwise healthy young adults remains unclear. Young adults with mild symptoms of SARS‐CoV‐2 (8M/8F, age: 21 ± 1 years, BMI: 23.5 ± 3.1 kg·m^−2^) underwent monthly transthoracic echocardiography (TTE) and testing of circulating cardiac troponin‐I for months 1–6 (M1–M6) following a positive polymerase chain reaction test to better understand the acute effects and post‐acute sequelae of SARS‐CoV‐2 on cardiac structure and function. Left heart structure and ejection fraction were unaltered from M1–M6 (*p* > 0.05). While most parameters of septal and lateral wall velocities, mitral and tricuspid valve, and pulmonary vein (PV) were unaltered from M1–M6 (*p* > 0.05), lateral wall *s′* wave velocity increased (M1: 0.113 ± 0.019 m·s^−1^, M6: 0.135 ± 0.022 m·s^−1^, *p* = 0.013); PV *S* wave velocity increased (M1: 0.596 ± 0.099 m·s^−1^, M6: 0.824 ± 0.118 m·s^−1^, *p* < 0.001); the difference between PV *A* wave and mitral valve (MV) *A* wave durations decreased (M1: 39.139 ± 43.715 ms, M6: 18.037 ± 7.227 ms, *p* = 0.002); the ratio of PV *A* duration to MV *A* duration increased (M1: 0.844 ± 0.205, M6: 1.013 ± 0.132, *p* = 0.013); and cardiac troponin‐I levels decreased (M1: 0.38 ± 0.20 ng·ml^−1^, M3: 0.28 ± 0.34 ng·ml^−1^, M6: 0.29 ± 0.16 ng·ml^−1^; *p* = 0.002) over time. While young adults with mild symptoms of SARS‐CoV‐2 lacked changes to cardiac structure, the subclinical improvements to cardiac function and reduced inflammatory marker of cardiac troponin‐I over 6 months following SARS‐CoV‐2 infection provide physiologic guidance to post‐acute sequelae and recovery from SARS‐CoV‐2 and its variants using conventional TTE.

## Graphical abstract

This study sought to investigate the impact of SARS‐CoV‐2 infection on cardiac structure and function over 6 months of recovery in young adults. Alterations to PV *S* wave velocity, durations of PV and MV *A* waves, the ratio of PV *A* wave duration to MV *A* wave duration, and cardiac troponin‐I levels were observed over the study period, suggesting favorable, albeit prolonged, myocardial changes over the 6‐month recovery period from SARS‐CoV‐2 infection in young adults.

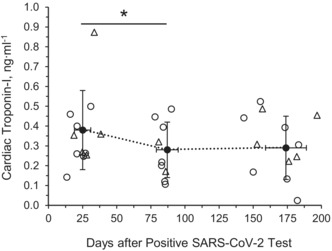

## 1 INTRODUCTION

The severe acute respiratory syndrome coronavirus‐2 (SARS‐CoV‐2) became a public health emergency of international concern in March 2020 (Habas et al., 2020). SARS‐CoV‐2 infection targets multiple organ systems, notably the respiratory and cardiac systems (Capotosto et al., 2020). SARS‐CoV‐2 infiltrates human tissues by binding to the angiotensin‐converting enzyme 2 (ACE2) receptor, which is present in the lungs, small intestine, vasculature, smooth muscle cells, and abundantly in the myocardium (Azevedo et al., 2021; Babapoor‐Farrokhran et al., 2020). Previously, SARS‐CoV‐1 infection resulted in the downregulation of ACE2 in the myocardium and leading to inflammation and cardiac dysfunction (Babapoor‐Farrokhran et al., 2020; Oudit et al., 2009).

While other coronaviruses were observed to have negative implications for the myocardium (Alhogbani, 2016; Farshidfar et al., 2021; Oudit et al., 2009), SARS‐CoV‐2 appears to yield more severe cardiac complications when compared with other human coronaviruses, such as SARS‐CoV‐1, even among young otherwise healthy adults (Kim et al., 2020; Rajpal et al., 2021; Shah et al., 2020). These manifestations may present acutely or exacerbate during post‐acute sequalae, where symptoms are ongoing 12 weeks post‐initial infection (Babapoor‐Farrokhran et al., 2020; Raman et al., 2022), and cardiovascular effects abound 12 months following hospitalization with SARS‐CoV‐2 infection (Faria et al., 2022). Further, our group has previously identified vast dysfunction among the autonomic and vascular systems acutely following SARS‐CoV‐2 infection among young adults with mild symptoms (Malek et al., 2021; Stute, Stickford, Province, et al., 2021; Stute, Stickford, Stickford, et al., 2021; Szeghy et al., 2021), some of which can persist out to 6 months (Szeghy et al., 2022). These systemic physiologic perturbations may trigger cardiac complications (Bois et al., 2021; Evans et al., 2020; Shen & Zipes, 2014; Vrints et al., 2021) as well as mortality risk (Andreas et al., 2014; Barretto et al., 2009) which may present themselves long after initial symptoms of viral infection have waned (Puntmann et al., 2020).

Myocardial complications, such as myocarditis, heart failure, and arrhythmias, are a common extrapulmonary manifestation of SARS‐CoV‐2 infection (Chang et al., 2021; Evans et al., 2020). As a relatively low‐cost alternative to cardiac magnetic resonance imaging, transthoracic echocardiography is routinely used to assess myocarditis as well as other cardiac abnormalities (Cameli et al., 2020; Pirzada et al., 2020), and has revealed various manners in which SARS‐CoV‐2 may affect myocardial structure and function. Further, myocardial injury, most often identified via elevated cardiac troponin lets, is a critical health concern among patients infected with SARS‐CoV‐2 (Kehl et al., 2012; Sandoval et al., 2020) and is associated with elevated mortality risk, (Al Abbasi et al., 2020). Additionally, high levels of cardiac troponin are correlated with elevations in inflammatory and coagulation biomarkers, as well as more severe hypoxemia and respiratory illness in SARS‐CoV‐2 patients (Giustino et al., 2020). Certainly, elucidating the long‐term effects of SARS‐CoV‐2 on the myocardium is essential to providing guidance for healthy recovery, especially among those with mild symptoms who may not seek out medical attention for their infection despite recent evidence suggesting first‐year cardiovascular disease burden is substantial following SARS‐CoV‐2 infection (Xie et al., 2022). Further, longitudinal tracking of such potential effects is essential to identify if long‐term integrative physiologic dysfunction, such as those observed among the systemic vasculature (Ratchford et al., 2021; Szeghy et al., 2021), which may exacerbate underlying cardiac impairments and thus impact post‐acute sequelae caused by SARS‐CoV‐2.

Many studies have focused on high‐risk populations, such as the elderly, immunocompromised individuals, individuals with comorbidities, and those with more severe symptoms of SARS‐CoV‐2 infection. However, myocarditis following SARS‐CoV‐2 infection has been reported even in young and active adults, such as competitive athletes, with only mild symptoms (Malek et al., 2021; Rajpal et al., 2021). Yet, little is known regarding the time course of these potential effects and if underlying physiologic impairments may augment cardiac dysfunction long term. Thus, this study sought to longitudinally examine changes to cardiac structure and function following SARS‐CoV‐2 infection among young, relatively healthy adults who were not hospitalized and experienced only mild symptoms. Specifically, we aimed to investigate the impact of SARS‐CoV‐2 infection on cardiac structure and function during months 1–6 (M1–M6) following SARS‐CoV‐2 infection in young adults. Due to the prevalence of myocardial inflammation in SARS‐CoV‐2 patients, we hypothesized subjects would demonstrate adverse subclinical changes in cardiac structure and function compared with reference values. Further, we expected such parameters to improve from M1 to M6 after SARS‐CoV‐2 infection in these young adults. Finally, we hypothesized that the circulating biomarker of cardiac injury, cardiac troponin‐I, would decrease from M1 to M6 after SARS‐CoV‐2 infection in these young adults. Thereby, these data may be relevant to better understand post‐acute sequelae from SARS‐CoV‐2 in young adults for comparison with other SARS‐CoV‐2 infected including those hospitalized with severe infection (Faria et al., 2022), with viral variants across the disease severity spectrum, as well as with similar coronaviruses even after the COVID‐19 pandemic.

## 2 METHODS

### 2.1 Subjects

Subjects were relatively young and healthy adults free from chronic metabolic, cardiovascular, and pulmonary disease. Potential subjects were excluded if they were smokers, had any orthopedic limitations, or were hospitalized due to the virus. Female subjects were premenopausal and not pregnant or trying to become pregnant. Subjects tested positive for SARS‐CoV‐2 using a nasopharyngeal swab polymerase chain reaction (PCR) assay at a community testing site 3–4 weeks before the start of study testing. All procedures were approved by the Appalachian State University Institutional Review Board (IRB_20‐0304). Subjects provided informed consent, in accordance with the standards outlined by the Declaration of Helsinki, prior to testing.

### 2.2 Study procedures

Following a positive PCR test for the SARS‐CoV‐2 virus, subjects came to the laboratory for a total of five visits. During the first study visit, subjects completed a health history questionnaire, detailing personal and family medical history and any medication usage. Subjects arrived for testing in a fasted state, having abstained from food for at least 4 h, caffeine for 12 h, and from exercise and alcohol for 24 h before testing. Study procedures were ordered similarly between subjects. Subjects returned to the laboratory once every approximately 4 weeks for additional testing through M6.

### 2.3 COVID‐19 symptom severity survey

Subjects were asked to rank their symptoms on each of the five visits of study testing. On a scale of 0–100 of increasing severity, subjects ranked their symptoms of chest pain, chills, diarrhea, dizziness or vertigo, dry cough, dry eyes, dry mouth, fatigue, fever (>37.9°C), headache, lack of appetite, loss of smell or taste (anosmia), muscle or body aches, nasal congestion or runny nose, nausea or vomiting, shortness of breath, difficulty breathing, dyspnea, sore joints, or sore throat. Mild symptoms were classified as a symptom severity score of 0–33, moderate 34–66, and severe 67–100. The total number of symptoms each subject experienced, as well as the severity score they reported for each present symptom, was recorded. The severity score was then divided by the number of symptoms to yield an average symptom severity score for each subject, which was then totaled and averaged for each visit, as previously described (Stute et al., 2021). Additionally, participants subjectively provided their recent physical activity level each month based on days each week and duration of physical activity.

### 2.4 Transthoracic echocardiography

Before echocardiography was performed by a single trained sonographer, subjects rested in a supine position for at least 20 min. Doppler ultrasound was used to perform transthoracic echocardiography (GE Logiq eR7 and 3Sc‐RS transducer, GE Medical Systems, Milwaukee, WI), analyzing subjects' cardiac structure and function using the parasternal long axis and apical four‐chamber views of the heart and pulsed‐wave tissue Doppler in the apical four‐chamber view. These views have been described thoroughly in other publications (Mohamed et al., 2010). The parasternal long‐axis view was utilized to measure diameters, wall thickness, and calculate mass. The apical four‐chamber view was utilized to obtain volumes, ejection fraction, mitral valve, tricuspid valve, and pulmonary vein measures. Pulsed‐wave tissue Doppler imaging was used to measure peak myocardial tissue velocities and to determine septal and lateral wall velocities (Ho & Solomon, 2006).

### 2.5 Experimental measurements

At least 10 cardiac cycles were recorded, and five cardiac cycles were selected to be analyzed for each variable based on image quality. A single, trained sonographer performed all analyses in random subject/visit order, and results were not populated until all analyses were performed to prevent bias.

The parasternal long‐axis view provided measures of diastolic interventricular septal wall thickness (IVS), left ventricular (LV) internal diameter (LVID), diastolic LV posterior wall thickness (LVPW), LV mass at end‐diastole, and left atrial (LA) diameter. End diastolic and systolic volume and ejection fraction were obtained using the biplane method of discs in the four‐chamber apical view.

Measures of septal and lateral wall velocities were obtained from the four‐chamber view, including septal and lateral wall *s′*, *e′*, and *a′* values, as well as septal and lateral wall *E*/*e′* ratios.

The four‐chamber view also provided measures for mitral valve (MV) function: MV *E* wave velocity, *A* wave velocity, *E*/*A* ratio, deceleration time, and MV *A* wave duration. Tricuspid valve (TV) *E* and *A* wave velocities, *E*/*A* ratio, deceleration time, and pressure half time (PHT) were derived from the four‐chamber view.

Additionally, pulmonary vein (PV) measurements, including systolic pulmonary vein (PV *S*) and diastolic pulmonary vein (PV *D*) wave velocity, *S*/*D* ratio, *A* wave (PV *A*) duration, PV *A* duration and MV *A* duration difference, and PV *A* to MV *A* duration ratio were obtained using the four‐chamber view.

### 2.6 Circulating cardiac troponin‐I

Antecubital vein serum blood samples were collected following echocardiography measurements at M1, M3, and M6, on a subset of subjects who consented to blood draws (M1: 7M/4F, M3: 7M/3F, and M6: 7M/3F). Enzyme‐linked immunosorbent assay kits (CALBIOTECH: #TI051C) were used to quantitatively determine cardiac troponin‐I following manufacturer's instructions.

### 2.7 Statistical analysis

Statistical analysis was performed using commercially available software (Armonk, NY; SAS Version 9.4, Cary, NC). Each continuous variable was checked for normality using Kolmogorov–Smirnov tests. Normality was confirmed visually with Q‐Q plots. Studentized residuals falling outside of three standard deviations were considered outliers and removed from analysis. Linear mixed models were used to determine main effects of time (month) for all outcome variables. Tukey–Kramer post hoc tests were used where significant effects were observed. Normative data were used for comparisons across time using repeated one sample *z*‐tests accounting for repeated measures, since a prospective time control group was not possible during the active COVID‐19 pandemic with a fluid situation of emerging viral variances, false‐negative testing of potential control subjects, and vaccination status which would have made control subjects difficult to recruit, validate as control subjects, and retain throughout the study duration (Pecoraro et al., 2022; Szeghy & Ratchford, 2022). Statistical significance was set at *p* < 0.05. Data are reported as mean ± standard deviation (SD).

## 3 RESULTS

### 3.1 Subject characteristics

Subject characteristics for the 16 subjects (8M/8F) are presented in Table 1. Transthoracic echocardiography was performed over 6 months, with 16 subjects (8M/8F) for M1 (25 ± 5 days since positive test), 16 subjects (8M/8F) for M2 (57 ± 7 days since positive test), 12 subjects (7M/5F) for M3 (87 ± 8 days since positive test), 13 subjects (7M/6F) M4 (119 ± 13 days since positive test), and 12 subjects (7M/5F) for M6 (174 ± 15 days since positive test). Subject attrition led to combining of the subjects' final visit and labeled M6, with three (3M) subjects completing only M5, and nine (4M/5F) subjects completing their last visit at M6. Six female subjects reported hormonal contraceptive use in M1 and M2, four female subjects in M3 and M4, and three female subjects in M6. Three subjects were taking selective serotonin reuptake inhibitors (SSRIs) in M1, M2, and M3, and only one subject reported use of SSRIs in M4 and M6.

**TABLE 1:** Subject characteristics

Subject characteristics	Month 1 (25 ± 5 days)	Month 2 (57 ± 7 days)	Month 3 (87 ± 8 days)	Month 4 (119 ± 13 days)	Month 6 (174 ± 15 days)
Subjects (n, M/F)	16 (8 M/8F)	16 (8 M/8F)	12 (7 M/5F)	13 (7 M/6F)	12 (7 M/5F)
Age, years	21 ± 1	21 ± 1	22 ± 1	21 ± 1	21 ± 1
Height, cm	176 ± 10	176 ± 9	176 ± 11	176 ± 10	176 ± 10
Weight, kg	72 ± 11	73 ± 12	73 ± 7	73 ± 12	74 ± 12
Body mass index, kg·m^−2^	23.5 ± 3.1	23.7 ± 3.09	23.6 ± 2.3	23.7 ± 3.1	23.7 ± 2.9
Resting supine heart rate, bpm	64 ± 11	60 ± 10	61 ± 8	61 ± 8	57 ± 9
Physical activity					
Frequency, days per week	4 ± 1	4 ± 1	4 ± 1	4 ± 1	4 ± 1
Duration, min per day	41 ± 12	41 ± 12	39 ± 13	40 ± 13	39 ± 13
Average symptom severity score	17.9 ± 12.3	15.1 ± 13.5	9.5 ± 10.5	7.2 ± 9.6	0.2 ± 0.6
Number of symptoms	3.7 ± 2.0	1.9 ± 1.3	1.3 ± 1.6	0.9 ± 1.1	0.3 ± 0.9
Female contraceptive use	6	6	3	4	3

*Note*: Data are mean ± SD.

Abbreviations: M, male; F, female.

Four subjects (3M/1F) received either one or both doses of the SARS‐CoV‐2 vaccine during the study (three Moderna and one Pfizer). One female participant received her first dose of Moderna between M4 and M6. Two male participants received their first dose between M2 and M3, and second dose between M4 and M5 (one Moderna and one Pfizer). One other male participant received his first dose of Moderna between M1 and M2, and second dose between M2 and M3. Vaccinated subjects did not exhibit noticeable differences in measured outcomes compared with the unvaccinated subjects in this investigation. Further, participants' study visits were scheduled at least 2 weeks following participants' previous vaccine dates to diminish the impact of vaccination effects on outcome variables, a design previously utilized in vaccination studies (Lind et al., 2012). These vaccinated participants' results were generally similar to all other unvaccinated participants' results. Therefore, participants were not separated by vaccination status.

### 3.2 Symptom severity

Average symptom severity survey scores are displayed in Table 1. All symptoms in the SARS‐CoV‐2 subjects were mild (score < 34).

### 3.3 Myocardial structure

The IVS thickness during diastole tended to change over time (*p =* 0.052; Table 2). There was no main effect of time for LVID during diastole (*p* = 0.610) or systole (*p* = 0.520), systolic LVID indexed to body surface area (*p* = 0.526), end diastolic (*p* = 0.438) or systolic volume (*p* = 0.874), LVPW during diastole (*p* = 0.432), LV mass (*p* = 0.884) or indexed to body surface area (*p* = 0.870), or left atrium diameter during diastole (*p* = 0.484). Similarly, there was no main effect of time for ejection fraction (*p* = 0.982; Table 2). When compared with reference values, IVS thickness during diastole (M1: *p* < 0.001, M2: *p* < 0.001), LVID during systole (M1: *p* = 0.010, M2: *p* < 0.001, M3: *p* = 0.010, M4: *p* < 0.001, M6: *p* < 0.001), end systolic volume (M1: *p* < 0.001, M2: *p* < 0.001, M3: *p* < 0.001, M4: *p* < 0.001, M6: *p* < 0.001), LVPW (M1: *p* < 0.001, M2: *p* < 0.001, M3: *p* < 0.001, M4: *p* < 0.001, M6: *p* < 0.001), LV mass (M1: *p* < 0.001, M2: *p* < 0.001, M3: *p* < 0.001, M4: *p* < 0.001, M6: *p* < 0.001), and LV mass index (M1: *p* < 0.001, M2: *p* < 0.001, M3: *p* < 0.001, M4: *p* < 0.001, M6: *p* < 0.001) were significantly different at the respective timepoints.

**TABLE 2:** Myocardial structure

Myocardial structure	Reference values	Month 1 (25 ± 5 days)	Month 2 (57 ± 7 days)	Month 3 (87 ± 8 days)	Month 4 (119 ± 13 days)	Month 6 (174 ± 15 days)
Diastolic IVS thickness, cm	0.86 ± 0.16 (Kou et al., 2014)	0.92 ± 0.30*	1.01 ± 0.12*	0.92 ± 0.13	0.91 ± 0.10	0.88 ± 0.12
Diastolic LVID, cm	4.43 ± 0.48 (Kou et al., 2014)	4.45 ± 0.44	4.47 ± 0.57	4.56 ± 0.38	4.60 ± 0.51	4.70 ± 0.40
Systolic LVID, cm	2.99 ± 0.47 (Kou et al., 2014)	3.35 ± 0.35*	3.41 ± 0.64*	3.47 ± 0.24*	3.54 ± 0.32*	3.57 ± 0.37*
Systolic LVID index, cm·m^−2^	1.67 ± 0.26 (Kou et al., 2014)	1.80 ± 0.16	1.80 ± 0.26	1.86 ± 0.20*	1.88 ± 0.16*	1.89 ± 0.18*
End diastolic volume, ml	93.9 ± 27.0 (Kou et al., 2014)	113.3 ± 23.6	115.5 ± 26.2	123.6 ± 21.2	118.6 ± 28.6	128.1 ± 20.8
End systolic volume ml	34.3 ± 11.8 (Kou et al., 2014)	51.21 ± 18.98*	49.50 ± 14.76*	53.18 ± 15.17*	51.07 ± 18.20*	55.09 ± 13.05*
Diastolic LV posterior wall thickness, cm	0.88 ± 0.15 (Kou et al., 2014)	1.12 ± 0.15*	1.08 ± 0.15*	1.42 ± 0.13*	1.13 ± 0.20*	1.54 ± 0.22*
LV mass, g	126.8 ± 37.4 (Kou et al., 2014)	192.5 ± 45.2*	196.6 ± 56.8*	182.9 ± 46.3*	191.6 ± 44.0*	204.9 ± 57.8*
LV mass index, g·m^−2^	69.9 ± 17.5 (Kou et al., 2014)	102.0 ± 16.8*	106.4 ± 27.9*	97.7 ± 24.4*	100.9 ± 19.1*	107.18 ± 26.6*
Ejection fraction, %	63.6 ± 4.7 (Caballero et al., 2015)	55.4 ± 9.1	56.6 ± 8.3	56.8 ± 8.4	57.4 ± 7.8	56.7 ± 7.1
Diastolic LA diameter, cm	3.36 ± 0.43 (Kou et al., 2014)	2.65 ± 0.41	2.55 ± 0.33	2.70 ± 0.39	2.75 ± 0.36	2.74 ± 0.32

*Note*: Month 1 values were compared with reference values using *z*‐tests. Linear mixed models were used to examine the main effect of time. *z*‐test versus reference value. Data are mean ± SD.

Abbreviations: IVS, interventricular septal wall; LA, left atrial; LV, left ventricular; LVID, left ventricular internal diameter.

* *p* < 0.05.

### 3.4 Septal and lateral wall velocities

There was a significant main effect of time for lateral wall *s′* wave velocity (*p* = 0.013), wherein velocities were greater at M6 compared with M1 (*p* = 0.005), as displayed in Table 3. However, there was no main effect of time for septal wall *s′* wave velocity (*p* = 0.583), septal wall *e′* wave velocity (*p* = 0.915), septal wall *a′* wave velocity (*p* = 0.973), or the septal wall *E*/*e*′ ratio (*p =* 0.934; Table 3). Similarly, there was no main effect of time for lateral wall *e′* wave velocity (*p* = 0.710), lateral wall *a′* wave velocity (*p* = 0.762), or the lateral wall *E*/*e*′ ratio (*p* = 0.648; Table 3). When compared with reference values, septal *s′* wave velocity (M4: *p* = 0.020, *s*) and *e*′ wave velocity (M1: *p* < 0.001, M2: *p* = 0.005, M3: *p* = 0.030, M4: *p* = 0.025, M6: *p* < 0.001), septal *a*′ wave velocity (M1: *p* < 0.001, M2: *p* < 0.001, M3: *p* < 0.001, M4: *p* < 0.001, M6: *p* < 0.001), septal *E*/*e*′ (M1: *p* = 0.014, M2: *p* = 0.022), lateral *s′* wave velocity (M6: *p* < 0.001), and lateral *a*′ wave velocity (M1: *p* = 0.005, M2: *p* = 0.014, M3: *p* = 0.012, M4: *p* < 0.001) were significantly different at the respective timepoints.

**TABLE 3:** Septal and lateral wall velocities

Septal and lateral wall velocities measurement	Reference values	Month 1 (25 ± 5 days)	Month 2 (57 ± 7 days)	Month 3 (87 ± 8 days)	Month 4 (119 ± 13 days)	Month 6 (174 ± 15 days)
Septal *s*′ wave velocity, m·s^−1^	0.09 ± 0.01 (Caballero et al., 2015)	0.10 ± 0.02	0.09 ± 0.01	0.09 ± 0.01	0.10 ± 0.01^‡^	0.10 ± 0.01
Septal *e*′ wave velocity, m·s^−1^	0.12 ± 0.03 (Caballero et al., 2015)	0.15 ± 0.04^‡^	0.14 ± 0.02^‡^	0.14 ± 0.02^‡^	0.14 ± 0.02^‡^	0.14 ± 0.02^‡^
Septal *a*′ wave velocity, m·s^−1^	0.09 ± 0.02 (Caballero et al., 2015)	0.07 ± 0.01^‡^	0.07 ± 0.01^‡^	0.07 ± 0.01^‡^	0.06 ± 0.01^‡^	0.06 ± 0.01^‡^
Septal *E*/*e*′	6.90 ± 1.60 (Caballero et al., 2015)	5.54 ± 1.57^‡^	5.55 ± 1.18^‡^	5.86 ± 1.04	5.77 ± 0.88	5.76 ± 1.15
Lateral s′ wave velocity, m·s^−1^ ^†^	0.11 ± 0.02 (Caballero et al., 2015)	0.11 ± 0.02	0.12 ± 0.02	0.11 ± 0.02	0.12 ± 0.04	0.14 ± 0.02^‡^, *
Lateral *e*′ wave velocity, m·s^−1^	0.16 ± 0.03 (Caballero et al., 2015)	0.16 ± 0.03	0.16 ± 0.03	0.17 ± 0.02	0.16 ± 0.02	0.17 ± 0.02
Lateral *a*′ wave velocity, m·s^−1^	0.08 ± 0.02 (Caballero et al., 2015)	0.06 ± 0.02^‡^	0.07 ± 0.02^‡^	0.06 ± 0.02^‡^	0.06 ± 0.02^‡^	0.07 ± 0.01
Lateral *E*/*e*′	5.1 ± 1.3 (Caballero et al., 2015)	5.06 ± 1.32	5.17 ± 1.63	4.85 ± 0.58	5.14 ± 0.75	4.73 ± 0.66

*Note*: Month 1 values were compared with reference values using *z*‐tests. Linear mixed models were used to examine the main effect of time. Data are Mean ± SD. *a*′, mitral annulus velocity in late diastole; *E*, transmitral early peak velocity; *e*′, mitral annulus velocity in early diastole; *s*′, mitral annulus velocity in systole.

* Versus Month 1, *p* < 0.05.

^†^ Main effect for time, *p* < 0.05.

^‡^ *p* < 0.05, *z*‐test versus reference value.

### 3.5 Mitral valve

**FIGURE 1:**
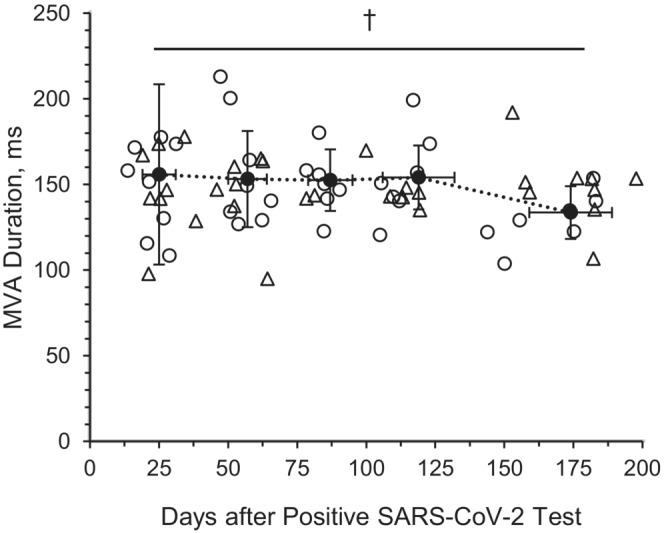
Mitral valve *A* wave (MV *A*) duration in young adults with SARS‐CoV‐2. Month 1: 25 ± 5 days post positive SARS‐CoV‐2 test (8M/8F); Month 2: 57 ± 7 days post positive SARS‐CoV‐2 test (8M/8F); Month 3: 87 ± 8 days post positive SARS‐CoV‐2 test (7M/5F); Month 4: 119 ± 13 days post positive SARS‐CoV‐2 test (7M/6F); and Month 6: 174 ± 15 days post positive SARS‐CoV‐2 test (7M/5F). Linear mixed models were used to determine main effects of time. Circle, male subject; triangle, female subject. ^†^Main effect of time, *p* < 0.05. Data are mean ± SD

There was a significant decrease over time for MV *A* wave duration (*p* = 0.027; Figure 1). However, there was no main effect of time for MV *E* (*p* = 0.973) or *A* wave velocities (*p* = 0.702), the MV *E*/*A* ratio (*p* = 0.546), and MV deceleration time (*p* = 0.849; Table 4). When compared with reference values, MV *A* wave velocity (M4: *p* = 0.018, M6: *p* = 0.049), MV *E*/*A* ratio (M4: *p* = 0.015), and MV *A* duration (M2: *p* = 0.025, M3: *p* = 0.018, M4: *p* = 0.026) were significantly different at the respective timepoints.

**TABLE 4:** Mitral valve

Mitral valve measurement	Reference values	Month 1 (25 ± 5 days)	Month 2 (57 ± 7 days)	Month 3 (87 ± 8 days)	Month 4 (119 ± 13 days)	Month 6 (174 ± 15 days)
MV *E* wave velocity, m·s^−1^	0.82 ± 0.16 (Caballero et al., 2015)	0.79 ± 0.17	0.79 ± 0.16	0.81 ± 0.11	0.82 ± 0.11	0.80 ± 0.10
MV *A* wave velocity, m·s^−1^	0.50 ± 0.13 (Caballero et al., 2015)	0.43 ± 0.06	0.53 ± 0.43	0.41 ± 0.09	0.40 ± 0.08^†^	0.40 ± 0.08^†^
MV *E*/*A* ratio	1.71 ± 0.52 (Caballero et al., 2015)	1.89 ± 0.45	1.94 ± 0.53	2.02 ± 0.32	2.14 ± 0.40^†^	2.08 ± 0.40
MV deceleration time, ms	213 ± 26 (Munagala et al., 2003)	222 ± 63	223 ± 73	225 ± 50	218 ± 56	220 ± 32
MV *A* duration, ms*	142 ± 15 (Munagala et al., 2003)	147 ± 26	153 ± 28^†^	152 ± 18^†^	151 ± 19^†^	131 ± 17

*Note*: Linear mixed models were used to examine the main effect of time. Data are Mean ± SD. Month 1 values were compared with reference values using *z*‐tests.

Abbreviations: *A*, transmitral late peak velocity; *E*, transmitral early peak velocity; MV, mitral valve.

* Main effect for time, *p* < 0.05.

^†^ *p* < 0.05, *z*‐test versus reference value.

### 3.6 Tricuspid valve

TV *E* wave velocity (*p* < 0.001), TV *A* wave velocity (*p* = 0.040), and TV PHT (*p* < 0.001) were higher at M1 than reference values. There was no main effect of time for TV *E* velocity (*p* = 0.998), TV *A* velocity (*p* = 0.098), TV *E*/*A* ratio (*p* = 0.254), TV deceleration time (*p* = 0.541), or TV PHT (*p* = 0.539; Table 5). When compared with reference values, TV *E* wave velocity (M1: *p* < 0.001, M2: *p* < 0.001, M3: *p* < 0.001, M4: *p* < 0.001, M6: *p* < 0.001), TV *A* wave velocity (M: *p* = 0.040), and TV PHT (M1: *p* < 0.001, M2: *p* < 0.001, M3: *p* < 0.001, M4: *p* < 0.001, M6: *p* < 0.001) were significantly different at the respective timepoints.

**TABLE 5:** Tricuspid valve

Tricuspid valve measurement	Reference values	Month 1 (25 ± 5 days)	Month 2 (57 ± 7 days)	Month 3 (87 ± 8 days)	Month 4 (119 ± 13 days)	Month 6 (174 ± 15 days)
TV *E* wave velocity, m·s^−1^	0.51 ± 0.08 (Pye et al., 1991)	0.61 ± 0.11*	0.62 ± 0.17*	0.63 ± 0.10*	0.62 ± 0.11*	0.62 ± 0.11*
TV *A* wave velocity, m·s^−1^	0.35 ± 0.09 (Pye et al., 1991)	0.41 ± 0.09*	0.37 ± 0.07	0.34 ± 0.03	0.37 ± 0.08	0.36 ± 0.07
TV *E*/*A* ratio	1.75 ± 0.67 (Pye et al., 1991)	1.56 ± 0.34	1.70 ± 0.36	1.77 ± 0.35	1.74 ± 0.28	1.74 ± 0.35
TV deceleration time, ms	120–229 (Zaidi et al., 2020)	233 ± 80	242 ± 62	258 ± 79	217 ± 49	220 ± 44
TV PHT, ms	51 ± 12 (Pye et al., 1991)	68 ± 23*	70 ± 18*	75 ± 23*	63 ± 17*	64 ± 13*

*Note*: Month 1 values were compared with reference values using *z*‐tests. Linear mixed models were used to examine the main effect of time. Data are Mean ± SD.

Abbreviations: *A*, trans‐tricuspid late peak velocity; *E*, trans‐tricuspid early peak velocity; PHT, pressure half time; TV, tricuspid valve.

* *p* < 0.05, *z*‐test versus reference value.

### 3.7 Pulmonary vein

**FIGURE 2:**
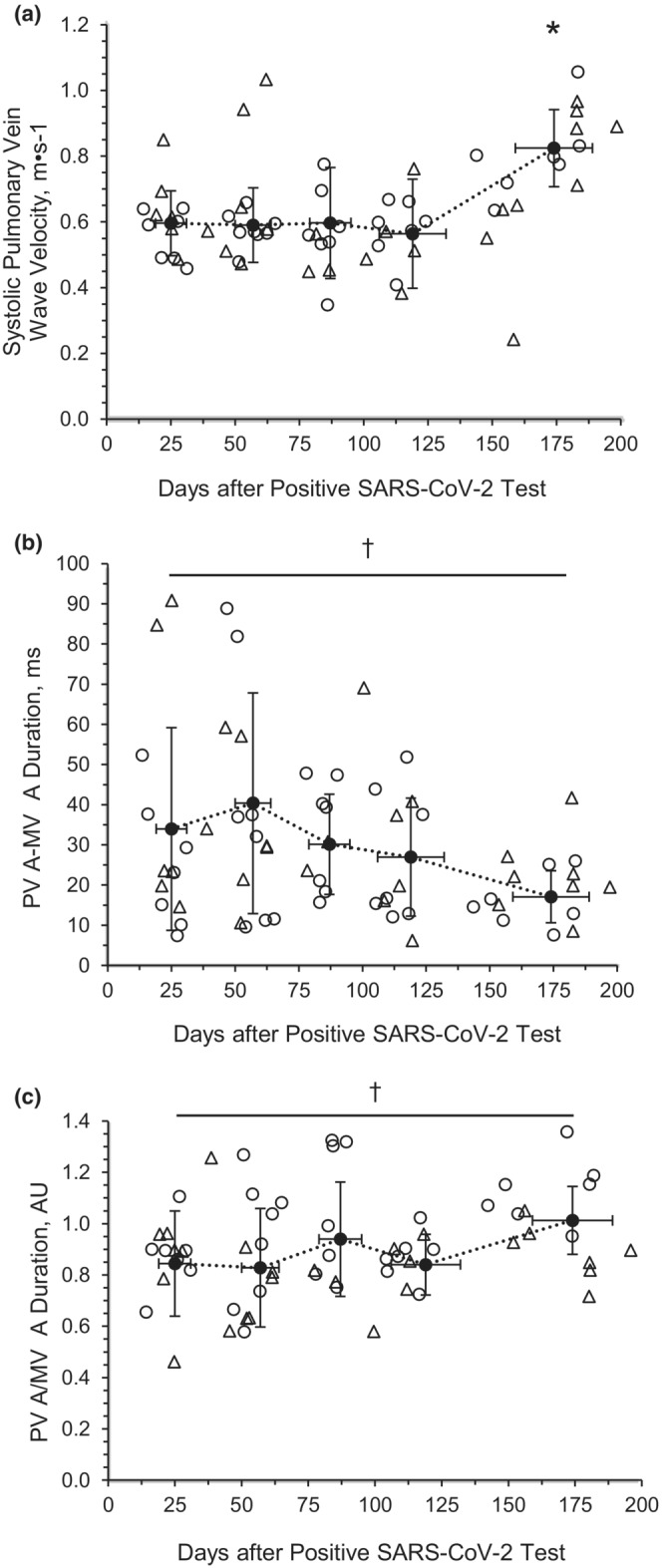
Pulmonary vein *S* wave velocity (PV *S*) (A), difference between pulmonary vein *A* wave duration and mitral valve A wave (PV *A* − MV *A*) duration (B), and ratio of pulmonary vein *A* wave duration and mitral valve *A* wave (PV *A*/MV *A*) duration (C) in young adults with SARS‐CoV‐2. Month 1: 25 ± 5 days post positive SARS‐CoV‐2 test (8M/8F); Month 2: 57 ± 7 days post positive SARS‐CoV‐2 test (8M/8F); Month 3: 87 ± 8 days post positive SARS‐CoV‐2 test (7M/5F); Month 4: 119 ± 13 days post positive SARS‐CoV‐2 test (7M/6F); and Month 6: 174 ± 15 days post positive SARS‐CoV‐2 test (7M/5F). Linear mixed models were used to determine main effects of time. Circle: male subject; triangle: female subject. ^†^Main effect of time, *p* < 0.05. *versus Month 1, *p* < 0.05. Data are mean ± SD

PV *D* wave (*p* = 0.002) was lower at M1 than reference values. There was a main effect of time for PV *S* wave velocity (*p* < 0.001), wherein velocities were greater at M6 compared with M1 (*p* < 0.0001; Figure 2a). There was no main effect of time for PV *D* wave velocity (*p* = 0.179) or the *S*/*D* ratio (*p* = 0.112; Table 6). However, there was a main effect of time for the difference between PV *A* duration and MV *A* duration (*p* = 0.001; Figure 2b) and the ratio of PV *A* duration to MV *A* duration (*p* = 0.009; Figure 2C). When compared with reference values, PV *S* wave velocity (M1: *p* = 0.018, M2: *p* = 0.014, M3: *p* = 0.040, M4: *p* = 0.038), PV *D* velocity (*p* < 0.001), PV *S*/*D* ratio (M6: *p* < 0.001), and PV *A* duration (M3: *p* = 0.042) were significantly different at the respective timepoints.

**TABLE 6:** Pulmonary vein

Pulmonary vein measurement	Reference values	Month 1 (25 ± 5 days)	Month 2 (57 ± 7 days)	Month 3 (87 ± 8 days)	Month 4 (119 ± 13 days)	Month 6 (174 ± 15 days)
Pulmonary vein *S* wave, m·s^−1^ ^†^	0.49 ± 0.12 (de Marchi et al., 2001)	0.40 ± 0.11^‡^	0.39 ± 0.09^‡^	0.40 ± 0.11^‡^	0.42 ± 0.12^‡^	0.48 ± 0.10*
Pulmonary vein *D* wave, m·s^−1^	0.64 ± 0.13 (de Marchi et al., 2001)	0.60 ± 0.10^‡^	0.59 ± 0.11	0.60 ± 0.17	0.56 ± 0.17	0.82 ± 0.12^‡^
Pulmonary vein *S*/*D* ratio	0.75 ± 0.23 (de Marchi et al., 2001)	0.64 ± 0.31	0.64 ± 0.32	0.64 ± 0.27	0.71 ± 0.38	0.56 ± 0.30^‡^
Pulmonary vein *A* duration, ms	118 ± 29 (de Marchi et al., 2001)	127 ± 26	125 ± 22	141 ± 30^‡^	128 ± 18	134 ± 18
A‐MV *A* duration, ms^†^	23 ± 34 (de Marchi et al., 2001)	34 ± 26	40 ± 27	29 ± 13	27 ± 15	17 ± 6

*Note*: Month 1 values were compared with Reference Values using *z*‐tests. Linear mixed models were used to examine the main effect of time. Data are Mean ± SD.

Abbreviations: *A*, pulmonary vein late peak velocity; *D*, diastolic; *S*, systolic; MV *A*, transmitral late peak velocity.

* Versus Month 1, *p* < 0.05.

^†^ Main effect for time, *p* < 0.05.

^‡^ *p* < 0.05, *z*‐test versus reference value.

### 3.8 Circulating cardiac troponin‐I

**FIGURE 3:**
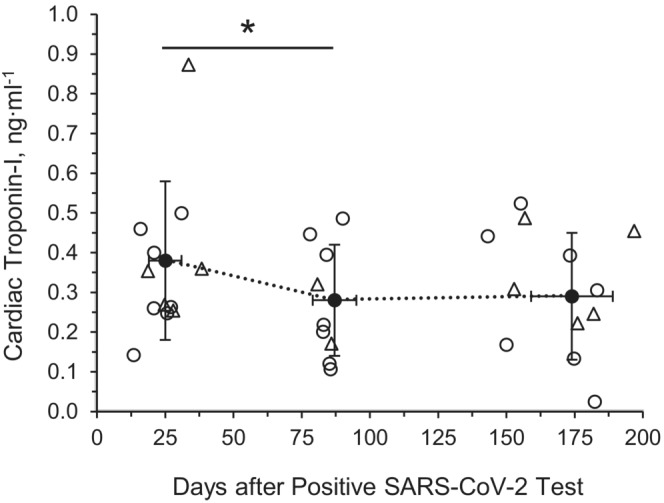
Circulating cardiac troponin‐I concentration in young adults with SARS‐CoV‐2. Month 1: 25 ± 5 days post positive SARS‐CoV‐2 test (7M/4F); month 3: 87 ± 8 days post positive SARS‐CoV‐2 test (7M/3F); Month 6: 174 ± 15 days post positive SARS‐CoV‐2 test (7M/3F). Linear mixed models were used to determine main effects of time. Circle: male subject; triangle: female subject. ^†^Main effect of time, *p* < 0.05. Data are mean ± SD

Cardiac troponin‐I levels were above 0.30 ng·ml^−1^ at M1, which is associated with myocardial injury (Arentz et al., 2020). A main effect of time for troponin‐I was observed (M1: 0.38 ± 0.20 ng·ml^−1^, M3: 0.28 ± 0.34 ng·ml^−1^, and M6: 0.29 ± 0.16 ng·ml^−1^; *p* = 0.002) with troponin‐I being reduced at M3 (*p* = 0.001) but not at M6 (*p* = 0.146) compared with M1, as shown in Figure 3. The coefficient of variation was 17.8%.

## 4 DISCUSSION

The purpose of this study was to examine the potential consequences of mild SARS‐CoV‐2 infection on cardiac structure and function in young, healthy adults, as subjects recovered from infection over 6 months. Our hypotheses were partially correct, as changes in several variables were observed throughout recovery, suggesting subjects experienced minor, subclinical changes to cardiac function following infection, requiring several months to improve. However, many variables remained unchanged over the course of our study, suggesting minimal effects to cardiac function following mild SARS‐CoV‐2 infection. Our major findings involve changes to parameters of blood flow through the mitral valve and pulmonary vein and increases in lateral wall *s′* velocity over the 6 months following infection. Importantly, circulating cardiac troponin‐I levels decreased from M1 to M3, indicating improvements to this biomarker for myocardial injury. However, there were largely no changes observed in cardiac structure or the tricuspid valve. Therefore, such parameters appear not to be adversely impacted by mild SARS‐CoV‐2 infection in healthy young adults. Together, these data provide clinically relevant observations for the myocardial recovery process among young adults with mild symptoms following infection with SARS‐CoV‐2 which may be relevant for those recovering from post‐acute sequelae of SARS‐CoV‐2 and similar coronaviruses.

### 4.1 Myocardial structure

Inflammation and thickening of the myocardium are notable cardiac malformations following SARS‐CoV‐2 infection (Babapoor‐Farrokhran et al., 2020; Chang et al., 2021; Faria et al., 2022; Pirzada et al., 2020). Myocardial inflammation may persist among individuals who recently recovered from SARS‐CoV‐2, even among those not hospitalized for SARS‐CoV‐2 (Puntmann et al., 2020). Further, myocarditis has even been observed in four out of 26 (15%) competitive college athletes following SARS‐CoV‐2 infection, of whom tend to not be obese nor comorbid which could otherwise exacerbate cardiac complications, with two of these subjects experiencing no symptoms (Rajpal et al., 2021). In this study, we observed diastolic IVS thickness tended to decrease in the first 6 months following SARS‐CoV‐2 infection, however, other measures of cardiac mass and ventricular volumes remained unaltered. Recently, subclinical elevations in left ventricular mass index were observed 1 year after hospitalization with SARS‐CoV‐2 in older adults (Faria et al., 2022), which may corroborate our findings of higher left ventricular mass index compared to reference values. These results may, therefore, provide subclinical evidence of myocardial inflammation following SARS‐CoV‐2 infection among young adults with mild symptoms. Surely, more work is needed to longitudinally track the recovery from myocarditis in patients with SARS‐CoV‐2 to better understand the potential long‐term impacts on the myocardium among a larger cohort and quite possibly using more sensitive techniques than transthoracic echocardiography.

### 4.2 Septal and lateral wall

Septal and lateral wall velocities provide insight into left ventricular function. In this study, we observed slight increases in lateral wall *s′* velocity, potentially indicating improvements in systolic function, as *s′* velocity is significantly correlated with LV ejection fraction (Park et al., 2010). Typical lateral wall *s′* values are 0.10 ± 0.03 m·s^−1^ for males and 0.10 ± 0.02 m·s^−1^ for females (Caballero et al., 2015), though, higher values for lateral wall *s′* may be expected in young adults. Systolic impairments after SARS‐CoV‐2 infection are rare, even among those with severe acute infection, affecting 9–11% of patients (Raman et al., 2022). Further, regional wall motion abnormalities and ischemia are correlated with reductions in lateral wall *s′* (Ho & Solomon, 2006). While increases in lateral wall *s′* may suggest systolic function may be improving over time, there were no observed changes in the speed of the relaxation of the lateral wall, as lateral wall *e*′ or *a′* values did not change over time. There were also no observed changes to septal wall velocities.

### 4.3 Mitral and tricuspid valve

Changes to blood flow through the mitral valve were observed, as the MV *A* wave duration decreased from M1 to M6 in this investigation. The mild reduction in the MV *A* wave duration over the 6 months of recovery from SARS‐CoV‐2 infection in young adults with mild symptoms may suggest a decrease in the duration of late diastole, which has been observed to be affected by reduced LV compliance and increased LV filling pressures in arterial hypertension (de Simone & Palmieri, 2001). However, this reduction in MV *A* duration also coincides with a reduction in heart rate which may account for the reduced late diastolic period. There were no significant changes in TV function over the course of recovery from mild SARS‐CoV‐2 infection. Previous studies have observed changes to right ventricle function, with RV dilation and dysfunction being the most common echocardiographic abnormalities noted following SARS‐CoV‐2 function (Argulian et al., 2020; Ghidini et al., 2021; Raman et al., 2022; Szekely et al., 2020), yet RV function was not directly measured in this investigation. Future studies are necessary to better understand the full extent of the longitudinal effects of mild SARS‐CoV‐2 infection on right‐sided cardiac function.

### 4.4 Pulmonary vein

The pulmonary veins, carrying oxygenated blood from the pulmonary circulation to the heart, can become damaged and lead to pulmonary edema or pleural effusion following viral infection (Hooper et al., 2001; Smiseth, 2015), and may be an indication of myocardial dysfunction. In this investigation, there was an increase in PV *S* wave velocity to become more similar to reference values, which may be indicative of LA relaxation and/or increased RV contractility (Smiseth, 2015; Tabata et al., 2003), which previous reports suggest may be impaired by moderate‐to‐severe SARS‐CoV‐2 infection (Argulian et al., 2020; Capotosto et al., 2020). Meanwhile, slight changes in MV *A* duration and PV *S* velocity may suggest slight improvements to LA function. However, with normal LA size, normal MV *A* velocity and normal lateral and septal *a*′ wave velocities, there may not be a strong indication of LA dysfunction among these young adults with mild symptoms of SARS‐CoV‐2 infection. Thus, the observed increases in the PV *S* wave velocity over time could extend the current understanding of the severity of RV alterations among young adults with mild symptoms of SARS‐CoV‐2 infection. Furthermore, differences of more than 30 ms between the PV *A* wave and MV *A* wave durations are indicative of elevated filling pressures, and thus diastolic dysfunction. In this study, we observed the difference in PV *A* and MV *A* wave durations to be greater than 30 ms at M1, which then slightly decreased to values below 30 ms across the 6 months of recovery, possibly improving this index of diastolic function.

### 4.5 Circulating cardiac troponin‐I

Elevations in cardiac troponin‐I are common among various respiratory infections, including influenza (Kwong et al., 2018), SARS‐CoV‐1, and MERS‐CoV (Madjid et al., 2020). In those with SARS‐CoV‐2, observed elevations in cardiac troponin‐I may be severity‐dependent (Lippi et al., 2020; Zhou et al., 2020). In this investigation, we observed decreases in circulating troponin‐I levels from M1 to M3 following SARS‐CoV‐2 infection among young adults with mild symptoms. The higher levels at M1 potentially suggest acute elevations in circulating troponin‐I due to viral infection among young adults which may resolve quickly, as has been suggested by others among hospitalized individuals with SARS‐CoV‐2 (Faria et al., 2022).

### 4.6 Limitations

We recognize several limitations in this investigation. Attrition decreased our sample size throughout the study, which is a common limitation of longitudinal investigations. However, several of our nonstatistically significant findings are the first of their kind and still warrant clinical consideration. The lack of baseline data for subjects prior to their contraction of SARS‐CoV‐2 also limits our interpretation of the observed changes, although this timepoint would be quite difficult to collect in this real‐world, observational investigation. The use of prospective control subjects during the active COVID‐19 pandemic has been contentious; therefore, normative data were used for comparison, and the subjects with SARS‐CoV‐2 were used to control for intraindividual variability in echocardiographic parameters. While we did not control oral contraceptive use, medication usage was maintained throughout the study. Further, hormonal contraceptives do not appear to impact smooth muscle function in the microvasculature or vascular structure and may not have a significant impact on cardiac structure and function (George et al., 2000; Williams & MacDonald, 2021). While the changes observed in this investigation may be subclinically relevant, we recognize the variability associated echocardiography may account for some of the changes observed in this investigation which should be corroborated by future investigations. Overall, our findings are strengthened by our repeated measures design and warrant clinical attention, although we recognize that larger cohort studies among varying illness severity and diseased populations are needed to extend these findings.

## 5 CONCLUSION

Our results indicate infection with mild SARS‐CoV‐2 causes a lack of change to cardiac structure and minimal changes to cardiac function, as most measures did not change significantly over time and remained similar to reference values. Nevertheless, our data suggest that there were some subclinical alterations to functional and structural cardiac variables in young adults. Further, significant decreases in circulating cardiac troponin‐I over time suggest a slight decrease in inflammation and myocardial improvement. While our cohort consisted of otherwise healthy, young adults devoid of preexisting cardiometabolic or pulmonary disease, these results could potentially be more problematic for those with underlying comorbidities which may be associated with post‐acute sequelae caused by SARS‐CoV‐2. Together, these results would provide necessary and thorough insight into the long‐term impact of SARS‐CoV‐2 infection on cardiac structure and function among young adults with mild symptoms and provide insight for individuals with post‐acute sequelae of SARS‐CoV‐2 or similar coronavirus infections.

## AUTHOR CONTRIBUTIONS

Jonathon L. Stickford, Abigail S. L. Stickford, and Stephen M. Ratchford conceived and designed research; Sophie S. Osada, Rachel E. Szeghy, Nina L. Stute, Valesha M. Province, Marc A. Augenreich, Jonathon L. Stickford, Abigail S. L. Stickford, and Stephen M. Ratchford performed experiments; Sophie S. Osada and Stephen M. Ratchford analyzed data; Sophie S. Osada, Rachel E. Szeghy, Nina L. Stute, Valesha M. Province, Marc A. Augenreich, Andrew Putnam, Jonathon L. Stickford, Abigail S. L. Stickford, Gregory J. Grosicki, and Stephen M. Ratchford interpreted results of experiments; Sophie S. Osada, Rachel E. Szeghy, and Stephen M. Ratchford prepared figures; Sophie S. Osada, Rachel E. Szeghy, and Stephen M. Ratchford drafted manuscript. All authors edited and revised manuscript. All authors have read and approved the final version of this manuscript and agreed to be accountable for all aspects of the work in ensuring that questions related to the accuracy or integrity of any part of the work are appropriately investigated and resolved. All persons designated as authors qualify for authorship, and all those who qualify for authorship are listed.

## FUNDING INFORMATION

This study was partially supported by an Internal COVID‐19 Research Cluster Award at Appalachian State University.

## ETHICS STATEMENT

All procedures were approved by the Appalachian State University Institutional Review Board (IRB_20‐0304) in accordance with the ethical standards described by the Declaration of Helsinki. Prior to testing, experimental procedures were explained, both in writing and verbally, and participants provided written informed consent.

## CONFLICTS OF INTEREST

The authors have no competing interests to declare.
